# Adjunct primer for the use of national comprehensive cancer network guidelines for the surgical management of cutaneous malignant melanoma patients

**DOI:** 10.1186/1477-7819-10-54

**Published:** 2012-04-06

**Authors:** Edibaldo Silva

**Affiliations:** 1 Division of Surgical Oncology Department of Surgery, University of Nebraska Medical Center, 984030 Nebraska Medical Center, Omaha, NE 68198-4030, USA

**Keywords:** Malignant melanoma, Surgical management, NCCN guidelines, Treatment outcomes

## Abstract

Recently, a Surveillance Epidemiology and End Results (SEER) survey of melanoma patterns of care by the Mayo Clinic, Scottsdale showed remarkable deviations from best practice patterns throughout the country. The study, which analyzed the SEER records of 35,126 stage I to III cutaneous malignant melanoma patients treated from 2004 to 2006, showed that adherence to National Comprehensive Cancer Network (NCCN) therapeutic resection margins occurred in less than 36% of patients. Similarly, considerable variation in the quality of melanoma care in the United States when assessed using 26 quality indicators drawn by a panel of melanoma experts was independently reported. These observations underscore the significant lack of adherence to published best practice patterns reflected by the NCCN guidelines. The untoward effects of these variations in practice pattern can have an inordinate impact on the survival of melanoma patients in whom long term outcomes are affected by the adequacy of surgical management. Thin malignant melanoma is curable; however, thick or node positive melanoma is often incurable. This outcome is determined not only by the stage at presentation but by the use of best practice patterns as reflected in current NCCN cutaneous melanoma practice guidelines.

## Review

Recently, a Surveillance Epidemiology and End Results (SEER) survey of melanoma patterns of care by the Mayo Clinic, Scottsdale showed remarkable deviations from best practice patterns throughout the country. The study, which analyzed the SEER records of 35,126 stage I to III melanoma patients treated from 2004 to 2006, showed that adherence to National Comprehensive Cancer Network (NCCN) therapeutic resection margins occurred in less than 36% of patients [1]. In as many as 11% of patients the initial diagnostic excisional biopsy was the only surgical resection undertaken. Approximately 40% of these patients were eligible for sentinel node biopsy (SNB) based on their micro-staging. However, only 53% of these patients ever had the SNB performed. Among those whose SNB was positive, 27% never had a therapeutic completion node dissection (CLND). These observations underscore the significant lack of adherence to published best practice patterns reflected by the NCCN guidelines. These guidelines are widely available on-line and remain the standard of care. Why is it that they are not universally utilized?

The departure from NCCN standards could be ascribed to the fact that most melanoma patients are not treated by trained melanoma specialists. Cursory review of the lengthy and thorough NCCN guidelines by episodic melanoma practitioners may be part of the problem. This was suggested by a recent publication by Erickson *et al. *(2008) in the community hospital setting where they observed that compliance with NCCN melanoma guidelines was suboptimal among non-surgical oncologists when compared to surgical oncologists [2]. In an attempt to formulate the NCCN standards into measurable quality indicators, Bilimoria (2009) reported that there remains "considerable variation in the quality of melanoma care in the United States" when assessed using 26 quality indicators drawn by a panel of melanoma experts [3]. For example, they report that only 25% of stage IB or II melanoma patients treated at Veteran Administration Hospitals in the United States had SNB compared to 60% at NCCN hospitals or 43% in the community hospital setting. Interestingly, most of the deviation in best practice patterns reported above result from the underutilization of a small subset of these guidelines [4]. However, it is noteworthy that non-adherence to this small subset of the guidelines results in the greatest deleterious impact on patient outcomes. Conversely, heightened attention to this subset may lead to significant improvement in clinical outcomes. More importantly, this primer should encourage even the inveterate practitioner to review the complete set of guidelines before embarking on the final treatment decision.

We propose to focus on the indispensable essentials, which account for the observed departure from adherence to the current NCCN standards for the surgical management of malignant melanoma. This selective approach could lead to decreased variation in the observed national practice patterns and improve outcomes. We fully underscore the importance of all of the NCCN guidelines but also recognize that the contemporary data suggests that they are not widely adopted. This adjunct to best practice patterns is amenable for use in academic and non-academic centers alike. This subset of the NCCN guidelines addresses the primary elements of melanoma diagnosis and treatment, which although notably deficient in the reported national studies, is employed by trained surgical and medical oncologists expert in the field. Focusing our attention on these streamlined guidelines may avert departures from NCCN best practice patterns. They address the following essential elements:

1) Diagnosis and biopsy

2) Microstaging and metastatic staging work up

3) Therapeutic resection margins

4) Indications for nodal staging with SNB

5) Therapeutic CLND

6) The role of metastasectomy in melanoma management

### Diagnosis and biopsy

All pigmented cutaneous lesions, in which melanoma is included in the clinical differential diagnosis, should undergo a complete full thickness excisional biopsy with minimal margins. For convenience, this can be done easily with a punch biopsy, which should include adjacent normal appearing skin [5]. Shave biopsy should be discouraged as superficial shaves may under stage the actual thickness or micro-staging of a lesion and confound subsequent treatment decisions, which are based solely on thickness, ulceration and mitotic activity. Melanoma can be fatal; therefore, all cutaneous lesions, which include melanoma as a possible diagnosis, should be biopsied.

### Micro-staging and metastatic staging work up

Although increasing annually in incidence, the majority of melanoma patients present with stage I disease. Only 16% of melanoma patients have nodal metastasis at diagnosis; therefore, for lesions with minimal likelihood of nodal metastasis no metastatic work up is indicated [6]. Patients with node negative thin (< 1 mm) melanoma and intermediate thickness melanoma (1 to 4 mm) without nodal involvement or specific symptoms suggestive of metastatic disease need not undergo a metastatic work up, computed tomography (CT) or CT/positron emission tomography (PET) or brain CT. In these patients (Stage IIa), the yield of positive findings on imaging is very low and those found to have a positive sentinel node could undergo metastatic work up after the SNB discloses the need for such a work-up prior to therapeutic CLND (Coit, 2011) [7]. Intermediate thickness melanoma with high risk features (ulceration, increased mitotic activity defined as greater than one mitotic figure per high power field) on micro-staging should likely undergo a metastatic work-up prior to SNB. No specific data exist to break down this recommendation by thickness; therefore, the decision is up to the treating physician. Likewise, thick melanoma (> 4 mm) is accompanied by substantial risk (> 60%) of distant metastases and should undergo a preoperative metastatic work-up. In the age of PET/CT, dedicated CT or magnetic resonance imaging (MRI) can be used to define inconclusive PET/CT findings, such as liver or brain involvement or elevated **lactate dehydrogenase **LDH.

### Therapeutic resection margins

Often the pathologist's report of a diagnostic biopsy of melanoma will include a statement regarding the presence or absence of tumor at the biopsy margins. This information is of no significance and can mislead many primary care physicians into not seeking additional surgical consultation. Therapeutic margins for melanoma are a function of the micro-staging thickness of the melanoma. Clear radial margins of 1 cm are required for all thin melanoma while 2 cm radial margins are recommended for all intermediate thickness lesions. These evidence-based recommendations have been designed to avoid a local recurrence, which is accompanied by a dismal five-year survival. Thick melanoma can be managed with 2 cm radial margins or greater if the lesion is associated with clinically evident satellitosis [7]. Melanoma *in situ *which has no metastatic potential should be excised with a 5 mm radial margin designed to remove all of the pre-malignant tissue to avoid a potential local invasive recurrence.

### Indications for nodal staging with SNB

Indications for nodal staging with SNB [8] in patients whose performance status is permissive of a subsequent therapeutic lymphadenectomy are:

A) All high risk thin melanoma (< 1 mm thick but associated with ulceration, increased mitotic activity or Clark level IV or V) should undergo SNB.

B) All melanoma thicker than 1 mm should undergo SNB.

### Therapeutic CLND

Unlike breast cancer, where contemporary evidence [9] has determined that lymphadenectomy for breast cancer is foremost a staging procedure, lymphadenectomy for cutaneous malignant melanoma is currently regarded as a therapeutic procedure. Morton has elegantly showed that all sentinel node micro-metastases progress to clinical macro-metastases [10]. Therefore, lymph node management for melanoma is different than that for breast cancer, a more familiar condition to many practicing physicians.

All patients with good performance status who have positive sentinel nodes should undergo a therapeutic CLND. Surgeons experienced in more complicated procedures, such as radical groin dissections or modified neck dissections, may be consulted in this setting. The presence of non-sentinel node involvement documented only by CLND is a significant adverse prognostic indicator. In a study by Coit *et al.*, 2,009 patients with positive non-sentinel node involvement had a median survival of 33 months while those with no non-sentinel node involvement had a median survival of 104 months [11]. These findings affirm the need for CLND in patients with a positive SNB. Yet, as reported by Wasif, 2010, Erickson, 2008 and more explicitly by McMasters, 2010, many have reported that only 50 to 69% of melanoma patients in the USA undergo a therapeutic CLND for positive SNB. In a preliminary review, among patients with histological positive SNB, the Multi-Center Lymph Node Trial demonstrated that patients undergoing a CLND showed a five-year survival advantage over those who did not (72.3% vs. 52%) [10,12]. In the Sunbelt Melanoma Trial, patients completing a CLND for positive SNB had a 67% five-year survival. Lastly, CLND affords excellent loco-regional control with nodal recurrence rates less than 4% compared with those not subject to a CLND for positive SNB [6]. In short, patients with pathologic stage III disease can be rendered free of tumor with aggressive loco-regional surgical therapy leading to improved survival.

### The role of metastasectomy in melanoma management

Recent progress in the targeted systemic therapy of stage IV melanoma is admittedly modest [13]. Thus, selective surgical intervention remains the mainstay for eradication of loco-regional recurrence at favorable metastatic sites. The success of aggressive metastasectomy hinges on the understanding of the biological behavior of melanoma and specifically the course of the illness on the individual patient. Without question, a long disease-free interval in excess of three to five years or more is a very important criterion for considering resection of a solitary metastatic focus. Solitary recurrences of the soft tissue and lung can be resected with excellent long term outcome. More modest outcomes can be seen in the resection of single brain metastasis and in selected patients even surgical intervention for liver metastases is useful [14,15].

In transit metastases defined as soft tissue, skin or dermal recurrences between the primary site and its anatomic nodal drainage basin are a frequent site of loco-regional recurrence. These are usually too numerous to resect, particularly when found in the extremities. In the extremities these are often expected sequelae in patients with thick melanoma with microscopic satellitosis and positive sentinel nodes who undergo a CLND. Prospective studies [16] from the Sidney Melanoma Unit in Australia have shown that isolated heated limb infusion as described by Thompson results in remarkable response rates in 85 to 95% of patients and eradication of disease defined as a complete remission in 50%. The technique is reproducible but calls for collaboration between surgeons, surgical oncologists, perfusionists and interventional radiologist for optimal outcomes.

In summary, even in the metastatic setting aggressive surgical management of selected stage IV patients can be very effective [14].

## Conclusions

Increased attention to the essential elements of current NCCN management guidelines for the surgical treatment of patients with cutaneous malignant melanoma can improve the care of most of these patients who present with early disease and improve outcomes. Variations in practice patterns resulting from the disuse of this subset of the guidelines account for most of the reported departure from best practice patterns. Additional recommendations to curb these practice variations, such as the inclusion of all melanoma patients in discussions at multi-disciplinary tumor boards, should already be in place. Yet, the sheer numbers of patients with early or favorable presentation does not permit the review of all of these cases, even in academic specialty centers. Another option is regionalization of care for malignant melanoma as has been done for complicated tumors, such as pancreatic or esophageal cancers. However, 1 in 74 Americans is diagnosed with cutaneous melanoma [17] and in the majority their management does not incur the potential morbidity and mortality inherent to the surgical management of pancreatic or esophageal cancer patients, which has resulted in the regionalization of their care. Therefore, regionalization would probably be impractical and costly.

The data suggest that the management of the newly diagnosed patient with malignant melanoma remains primarily a community-based surgical problem. An understating of the natural course of the illness and the role of surgical intervention in arresting its progression remains the most important determinant of satisfactory long term outcomes. In that vein, adherence to best practice patterns as elucidated by the NCCN standards should be paramount. Currently, reported variations in adherence to these best practice patterns in the surgical management of malignant melanoma can only have a negative impact on the overall survival of these patients. Yet, as shown herein, renewed attention to a key subset of admittedly underused NCCN guidelines for melanoma could lead to a significant improvement in outcomes. Approaches such as those suggested here may also enhance the use of the entire set of guidelines and narrow the reported variability in practice patterns.

## Abbreviations

**CLND**: completion node dissection; LCH: lactate dehydrogenase; NCCN: National Comprehensive Cancer Network; SEER: Surveillance Epidemiology and End Results; SNB: sentinel node biopsy.

## Competing interests

The author declares that they have no competing interests.

## Authors' contributions

ES, the author, is responsible for the composition and draft of this manuscript and has read and approved the final manuscript.
